# The Sooner, the Better: The Establishment of a Treatment Paradigm in Prostate Cancer

**DOI:** 10.3389/fphar.2017.00788

**Published:** 2017-10-31

**Authors:** Carlo Buonerba, Giuseppe Di Lorenzo

**Affiliations:** ^1^Department of Clinical Medicine and Surgery, University of Naples Federico II, Naples, Italy; ^2^Istituto Zooprofilattico Sperimentale Del Mezzogiorno, Portici, Italy

**Keywords:** prostate cancer, hormonal therapy, overall survival, safety, chemotherapy

Significant progresses have been recently made in the treatment of recurrent/metastatic hormone-sensitive prostate cancer. The findings reported in well conducted phase III trials (Sweeney et al., 2015; Duchesne et al., 2016; Fizazi et al., 2017; James et al., 2017) and in rigorous meta-analyses (Tucci et al., 2016; Rydzewska et al., 2017) consistently show that timing of hormonal and chemotherapy-based treatment initiation does have an impact on overall survival. In one meta-analysis including 2,951 patients with advanced hormone-sensitive prostate cancer, of whom 2,262 patients were metastatic, the addition of docetaxel to standard androgen-deprivation treatment for metastatic disease was associated with both longer OS (hazard ratio [HR]: 0.73; 95% confidence interval [CI], 0.60–0.90; *p* = 0.002) and progression-free survival (HR: 0.63; 95% CI, 0.57–0.70; *p* < 0.001) (Tucci et al., 2016). In another meta-analysis including 2,201 men with advanced hormone-sensitive prostate cancer (Rydzewska et al., 2017), the addition of abiraterone plus prednisone to standard androgen deprivation hormonal treatment was associated with improved OS (HR = 0.62, 95% confidence interval [CI] = 0.53–0.71, *p* < 0.001) and PFS (HR = 0.45, 95% CI = 0.40–0.51, *p* = 0.66 × 10–36), with no signal of a different survival benefits depending on performance status, Gleason sum score, or nodal status. Finally, a phase III trial (Duchesne et al., 2016) conducted in 293 men with advanced/recurrent hormone sensitive prostate cancer, 5-year overall survival in patients undergoing immediate vs. delayed androgen deprivation treatment was 91.2% (95% CI 84.2–95.2) in the immediate arm vs. 86.4% (95% CI 78.5–91.5) in the delayed arm (log-rank *p* = 0.047). In this trial, multiple triggers for treatment initiation were allowed, such as symptoms, PSA kinetics, or patient's or physician's preference. This advantage in 5-year overall survival was reported at the cost of a lower sexual activity and a higher rate of hormone-treatment-related symptoms at 6 and 12 months was, with no statistically significant differences in quality of life over the entire 5 years after randomization.

Taken together, these findings show that in patients with recurrent/advanced hormone-sensitive prostate cancer, systemic treatment has to be initiated as soon as possible in order to maximize its effectiveness without affecting quality of life (Duchesne et al., 2017). Similarly to the castration-resistant setting, abiraterone may seem a more attractive therapeutic option than docetaxel in men with metastatic, hormone-sensitive prostate cancer, as patients treated in this setting are often elderly and unfit for chemotherapy, with a meaningful risk of chemotherapy-associated death, as suggested by the LATITUDE authors (Fizazi et al., 2017). We believe that the safety risks associated with a prolonged (e.g., >1 year) treatment with abiraterone plus prednisone should be carefully considered in a real-life scenario. In the combined analysis of the STAMPEDE and LATITUDE trials, an approximate three-fold increase in grade III–IV acute cardiac (OR = 2.93, 95% CI 1.74–4.93, *p* < 0.001) and hepatic toxicity (OR = 3.09, 95% CI 2.12–4.50, *p* < 0.001) and an approximate 2-fold increase in grade III–IV vascular events (OR = 2.28, 95% CI 1.71–3.03, *p* < 0.001), mostly (≥90%) related to hypertension, were reported. These findings are worthy of attention if we consider that both patients in the LATITUDE and in the STAMPEDE trial with relevant heart conditions were excluded. In particular, patients with atrial fibrillation were not eligible for the LATITUDE trial. In the STAMPEDE trial, patients with any heart condition that could make him unfit for any of the study treatments according to the investigator's judgment were excluded. The majority of patients treated with abiraterone plus prednisone in the STAMPEDE trial did not have either diabetes (88%) or previous myocardial infarction (96%), or Congestive heart failure (100%), angina (96%) or even hypertension (58%), with only 14% of the population being smokers. Differently from such a selected population, elderly men in a real-life scenario are likely to have multiple cardiovascular risk factors or heart medical conditions such as atrial fibrillation (Kirchhof et al., 2017), which may translate into a worse treatment compliance and safety profile than those reported in the LATITUDE and STAMPEDE trials. Furthermore, in the combined analysis of these two trials, the size of benefit appeared greater in younger vs. older men (<70, ≥70: interaction HR = 1.54, 95% CI = 1.14–2.08, *p* = 0.005). Younger men are also more likely to tolerate chemotherapy as well.

When considering docetaxel and abiraterone in the hormone sensitive setting, treatment duration is one of the main issues, as it has implications in terms of financial costs, safety and quality of life. A 4-month course of docetaxel is likely to be as effective and as safe as a 33-month course of abiraterone, but also much less expensive. Whether a shorter, standardized course of abiraterone (e.g., 12 months) and whether a sequential or combination treatment of docetaxel plus abiraterone may further improve overall survival must be ascertained in the context of a randomized, controlled clinical trial.

At the present time, the only conclusion that can been drawn is that both docetaxel and abiraterone are able to extend overall survival in the hormone-sensitive setting to an apparently similar extent. Nevertheless, while the difference in cost and treatment duration strongly favors docetaxel, the safety profile of both treatment is acceptable, so we believe that treatment choices will mostly depend on reimbursement policies at a general level, and on patient's preference and medical history (e.g., heart conditions) at an individual level.

## Author contributions

All authors listed have made a substantial, direct and intellectual contribution to the work, and approved it for publication.

### Conflict of interest statement

The authors declare that the research was conducted in the absence of any commercial or financial relationships that could be construed as a potential conflict of interest.
